# University screening test at State University of Jakarta

**DOI:** 10.1186/1687-9856-2013-S1-P90

**Published:** 2013-10-03

**Authors:** Bina Akura, Lanny C Gultom

**Affiliations:** 1 Pediatric Endocrinology Division, Fatmawati Hospital; 2 Pediatric Nutrition and Metabolic Division, Fatmawati Hospital

**Keywords:** characteristics, obesity, insulin resistance

## Background

Insulin resistance is the greatest risk factor for the development of type 2 diabetes and is perhaps the greatest current health threat to our children. The prevalence of childhood obesity has more than double in the past 15 years in many regions of the world. The marked increase in pediatric obesity in the past decade has resulted in unprecedented increases in incidence of type 2 diabetes mellitus among children and adolescent.

## Aims

To describe the characteristics of obesity and insulin resistance among pre-university student at state university of Jakarta

## Methods

A cross sectional study was conducted during May 2012 – June 2012. Data was collected from adolescent before becoming university student. Subject was measured of body weight, height, body mass index, fasting insulin concentration (uM/mL) dan fasting glucose concentration (uM/mL). Subject was also calculated for homeostatic model assessment (HOMA). The HOMA cutoff point for diagnosing of insulin resistance is 3.16.

## Results

Of 390 adolescent enrolled at UNJ, before becoming university student, 20 subject were obese (5.1%). Most children were male (65%). From all subjects, mean body weight is 88.9±3.4 kg and mean height 165.8±2.2 cm. Body mass index of children with P95-97 was 30%, while BMI children with >P97 was 70%. Children with insulin resistance was 12 subjects (60%). Median of HOMA among all subjects is 4.04 (1.3 up to 13.7) and mean of BMI is 32.1±4.1. No significant difference between body weight and HOMA index (p=0.305) and also no significant difference between BMI and HOMA index (p=0.161).

## Conclusions

Most subjects were male. Most subjects were obese with mean body weight 88.9±3.4 kg and BMI 88.9±4.1. Only 60% subjects had insulin resistance. No significant difference between body weight, BMI and HOMA.

